# Assessment on Distributional Fairness of Physical Rehabilitation Resource Allocation: Geographic Accessibility Analysis Integrating Google Rating Mechanism

**DOI:** 10.3390/ijerph17207576

**Published:** 2020-10-18

**Authors:** Hui-Ching Wu, Ming-Hseng Tseng, Chuan-Chao Lin

**Affiliations:** 1 Department of Medical Sociology and Social Work, Chung Shan Medical University, Taichung 402367, Taiwan; graciewu@csmu.edu.tw; 2 Social Service Section, Chung Shan Medical University Hospital, Taichung 402367, Taiwan; 3 Department of Medical Informatics, Chung Shan Medical University, Taichung 402367, Taiwan; mht@csmu.edu.tw; 4 School of Medicine, Chung Shan Medical University, Taichung 402367, Taiwan; 5 Department of Physical Medicine and Rehabilitation, Chung Shan Medical University Hospital, Taichung 402367, Taiwan

**Keywords:** physical rehabilitation, elderly, geographic accessibility, resources allocation, spatial inequality, medical geology

## Abstract

Identifying and treating co-existing diseases are essential in healthcare for the elderly, while physical rehabilitation care teams can provide interdisciplinary geriatric care for the elderly. To evaluate the appropriateness of demand and supply between the population at demand and physical rehabilitation resources, a comparative analysis was carried out in this study. Our study applied seven statistical indices to assess five proposed methods those considered different factors for geographic accessibility analysis. Google ratings were included in the study as a crucial factor of choice probability in the equation for calculating the geographic accessibility scores, because people’s behavioral decisions are increasingly dependent on online rating information. The results showed that methods considering distances, the capacity of hospitals, and Google ratings’ integrally generated scores, are in better accordance with people’s decision-making behavior when they determine which resources of physical rehabilitation to use. It implies that concurrent considerations of non-spatial factors (online ratings and sizes of resource) are important. Our study proposed an integrated assessment method of geographical accessibility scores, which includes the spatial distribution, capacity of resources and online ratings in the mechanism. This research caters to countries that provide citizens with a higher degree of freedom in their medical choices and allows these countries to improve the fairness of resource allocation, raise the geographic accessibilities of physical rehabilitation resources, and promote aging in place.

## 1. Introduction

### 1.1. Physical Rehabilitation Resources and Active Aging

The World Health Organization (WHO) proposed a policy framework for active aging in 2002, emphasizing that active aging is a process wherein aging is guided by policies. By providing the elderly with the best opportunities in pursuit of health, social participation, and a safe environment, their quality of life can be effectively promoted [1]. Therefore, the crucial implication active aging is to help the elderly achieve the stage of successful aging. Phelan et al. [2] pointed out that the elderly believe successful aging involves the integration of multi-faceted health conditions, including physical, functional, psychological, and social abilities. In addition to medical services, social activities that increase mental flexibility and connection to support networks that strengthen health also promote the quality of life of the elderly. Due to the physical limitations of the elderly, the geographical accessibility of physical rehabilitation resources affects their ability to use community care resources and reflects fairness in the design of the resource allocation policy.

Identifying and treating co-existing diseases are essential in the healthcare for the elderly, while physical rehabilitation care teams can provide high-quality and interdisciplinary geriatric care for the elderly [3,4,5]. Board-certificated physiatrists are practitioners who complete their training in physical medicine and rehabilitation residency and pass the national examinations. They possess the professional knowledge to diagnose and treat many diseases of the elderly. Research has shown that with the intervention of physiatrists, the elderly enjoy better functional recovery from injuries and illnesses [6]. With the rapid growth of the elderly population, Taiwan is about to become a super-aged society. Every older person in Taiwan has the same health insurance. The current healthcare system in Taiwan, known as National Health Insurance (NHI), was instituted in 1995. NHI is a single-payer compulsory social insurance plan that centralizes the disbursement of healthcare funds. The system promises equal access to healthcare for all citizens, and the population coverage has reached 99% [7]. The National Health Insurance of Taiwan covers medical insurance for 99% of the population. People are free to choose from medical centers, community hospitals, and specialist clinics when they look for treatments. The integrated medical specialist teams led by physiatrists and supported by physiotherapists, occupational therapists, speech therapists, nurses, nutritionists, and orthotists can provide interdisciplinary physical rehabilitation care in appropriate environments with proper equipment and provide comprehensive care for the elderly [8,9].

### 1.2. Accessibility Assessment of Elderly Physical Rehabilitation Resources

Some studies addressed the perception of accessibility of elderly physical resources, such as reports by clinic managers versus actual accessibility in healthcare clinics for persons using wheelchairs [10], problems of access to primary care [11], people with physical disabilities feel they are experiencing difficulty accessing adequate and appropriate primary healthcare services [12]. According to these studies, the transportation factor is important for the elderly to access healthcare resources. Therefore, evaluating the appropriateness of the demand and supply between the population at demand and physical rehabilitation resource is important for policy-making. In a comprehensive review of the literature, studies that address a geographic accessibility assessment of elderly physical rehabilitation resources are rare.

A geographic accessibility assessment could provide a fair distribution in allocating healthcare resources [13,14,15,16,17,18,19,20,21,22,23,24]. Identifying and treating co-existing diseases are essential in healthcare for the elderly, while the accessibility of physical rehabilitation resources should be taken seriously.

Frail older adults can go to hospitals by their family’s vehicles or apply for the governmental rehabilitation bus service. Taiwan’s NHI provides a free rehabilitation bus service for those who have a handbook of physical and mental disabilities with moderate or above multiple disabilities including limbs, moderate or above visually impaired, vegetative (wheelchair accessible), and extremely severely disabled vital organs [25]. For those who could not pay the premium, the premium is fully subsidized for the households below the poverty line. Or, the NHI can refer those very poor persons to charitable organizations for help. The transportation for older adults is organized by families and the NHI. Therefore, income would not become the main obstacle of transportation, but the accessibility of resources would be an important issue for aging in place.

The discussion of fairness in the distribution of physical rehabilitation resources involves the degree of coordination between the population at demand and service supply as well as distance factors. For frail older adults who need regular and periodic physical rehabilitation, high-geographic accessibility is important to promote aging in place. The use of assessment methods for resource accessibility help examine whether the allocation of physical rehabilitation resources shows inequity due to regional differences. The author of this study attempted to employ geographic accessibility as the assessment method. The investigation analyzed and compared the results drawn from five types of geographic accessibility calculation methods examining the adequacy of physical rehabilitation resource allocation.

In this study, open data of 2020 were retrieved from Taiwan Academy of Physical Medicine and Rehabilitation, and its member list of board-certificated physiatrists and registered clinics was the supply points for the resources. People aged 65 and above in towns were held to be the population at demand. With the aforementioned data, the geographical accessibility of rehabilitation resources for the population at demand in towns were examined. For the presentation of analytical data, assessments focused on the data of county/city levels, which were aggregated from the data of town levels. Therefore, the counties/cities over the island that need to be prioritized for the improvement of resource accessibilities at physical rehabilitation points are pointed out in this study to present the problems in the appropriateness of demand and supply between geographical locations and the density distribution of population at demand. The research results are expected to turn into references for relevant administrative and management departments when they formulate resource allocation policies of rehabilitation resources.

The current distribution of the population at demand and physical rehabilitation resources, population at demand to physical rehabilitation resources ratio, and the service load of rehabilitation hospitals were examined in the study. The investigation helped consider how to increase the accessibilities of physical rehabilitation for the elderly by assisting them to look for treatment at clinics nearest to their homes and reduce traffic obstacles they may encounter to promote their health. The author explored the following issues:

1. The spatial distribution of the population at demand and number of physical rehabilitation resources in towns.
2. To carry out a comparative analysis on geographical accessibility scores of physical rehabilitation resources with five calculation methods based on different decision-making considerations and choice probabilities.
3. To suggest follow-up improvements of policies based on the differences in densities of physical rehabilitation resources in counties/cities.

## 2. Materials and Methods

### 2.1. Data Collection: Study Area and Datasets

The geographical area covered by the analysis in this study includes 19 counties/cities and 349 towns on the main island of Taiwan. Information about board-certificated physiatrists was retrieved from the open data of Taiwan Academy of Physical Medicine and Rehabilitation in 2020 [26]. Information about the population aged 65 and above in towns was retrieved from the database of Department of Household Registration, Ministry of the Interior, which was released in March 2020 [27].

The convenience of transportation is an important factor that determines senior citizens’ access to community care resources. However, to examine the differences in convenience of transportation in counties/cities, we will have to consider the types of vehicles, frequencies of running and travel time, fare policies, as well as fare subsidy policies of counties/cities. Due to the scarcity or low credibility of relevant data, it is infeasible to include such information in the analysis of road network data. In the evaluation of factors that affect geographic accessibility, the study took reference from the research method of Page et al. [28]. While retrieving data for the analysis of transportation influencing factors, the road network data in government open data representing actual route distances were adopted instead of the traditional map distances (the linear distance between two points) to reduce the error. As for map data, numerical maps were taken from the Ministry of Transportation and Communications [29]. The ArcGIS application, which adopts geographic information systems, was used to calculate geographic accessibility by a geography information system (GIS)-based network analysis. As the geographic accessibility analysis focused on the convenience of users’ mobility, if the data of supply points in the main island and outlying islands of Taiwan are mixed and assessed collectively, the issues in traffic and geographic distance will produce deviations in resource accessibility assessment. Therefore, the study area was limited to the main island of Taiwan.

To define the searching area of physiatrist resources, registered specialist clinics were listed and filtered in this study, according to the Taiwan Academy of Physical Medicine and Rehabilitation. As these resource data only list service units, we had to search for the addresses of every service unit before converting the addresses to coordinates by geocoding applications. Next, with the use of the geography information system (GIS), the latitudes and longitudes of the locations of every resource were positioned in the TWD97 2-degree transverse Mercator coordinate system. Cartographic visualization was employed to test the accuracy of every coordinated point and reduce location error. Finally, the cartographic data of physiatrist resources were produced. As of March 2020, there were 688 physiatrist service points in the main island of Taiwan, while there were 1140 physiatrists in total.

Preliminary investigations in this study indicated that there were 3,618,878 people aged 65 and above on the main island of Taiwan as of March 2020. As there were 1140 physiatrists in total, it means that for every 10,000 elderly people there were 3.15 physiatrists on average. As the number is close to the population of towns, the weighted center point of towns (generated by the weighed calculation of population in villages) would represent the center point of people in demand for resources.

### 2.2. Measuring Geographic Accessibility to Elderly Physical Rehabilitation Resources

The geographic accessibility of resources is a critical basis for considering resource allocation. A main method to analyze resource accessibility is to calculate the ratio of resources allocated (amount and spatial distribution) to the population at demand.

At present, Taiwan’s policy formulation relies on the regional average method in weighing medical resource accessibility. Taking each administrative region as a unit, the number of hospitals, medical personnel, and hospital beds per 10,000 (or per 100,000) people in the region is calculated and becomes a potential accessibility indicator for the framework of accessibility to medical resources [30]. In terms of the assessment of medical resources, the method assumes that the administrative region equal to the activity space where people utilize medical resources and distances does not bring about differences in the usage of medical resources within the region. However, patients can seek treatments by crossing into different administrative regions in reality. This characteristic of spatial mobility is not taken into consideration in the regional average method, and this is where problems arise [15]. The method was identified as method A0 in this study, with Equation (1) as follows:

(1) $$A_{i,0}=\;\frac{\sum_{j\in D_{i}}S_{j}}{P_{i}\;}$$

where *A_i_* is the geographic accessibility score of a town *i* and implies the average amount of supply point resources enjoyed by each person in demand in the region of the town *i*; $\sum_{j\in D_{i}}S_{j}$ represents the amount of supply point resources in region *i* of the town; *P_i_* represents the population at demand aged 65 or above in the region *i* of towns.

Luo and Wang [14] proposed the two-step floating catchment area method, which breaks the aforementioned limitations caused by setting administrative regions as activity areas. Not only does the research method consider the possibilities of cross-region healthcare utilization by people, but it also sets a reasonable range of seeking treatment and, in turn, assesses the spatial accessibility of medical resources. The two-step floating catchment area method is primarily divided into two stages [19,21,31]. In stage one, the service loads of each service provider of resources are calculated. In stage two, the ratios of resources that can be reached by each location of the population at demand are calculated to assess the geographic accessibility scores of resources [20].

The three-step floating catchment area method [32] is an advanced and improved search method derived from the two-step floating catchment area method. The new method evaluates different choice probabilities of the population at demand when people approach nearby locations of medical resources. The effects of hospitals’ capacities and travel distances on the utilization behavior of medical resources are specifically taken into consideration. The concept of this method is to calculate the probability of seeking treatment, which represents the probability of each patient to visit different hospitals through distance weighting and hospital capacities. The probability of seeking treatment is then used to estimate the average ability of the medical resource allocation of each hospital. In the same manner, with the hospitals’ capacities and distances from the served regions, the probability of each region in demand to visit different hospitals is calculated. According to the choice probabilities, the average ability of the medical resource allocation of each hospital will be allocated to the region in demand appropriately, wherein we obtain the distribution situation of geographical accessibility to medical resources in the research area.

The calculation of geographical accessibility proposed in this study originates from the calculus concepts of the three-step floating catchment area method. Equations (2)–(5) are as follows:

(2) $$A_{i,1}=\sum_{r=1\sim h}\underset{j\in D_{r}}{\sum }\frac{S_{j}*f\left(d_{ij}\right)}{\sum_{r=1\sim h}\sum_{k\in D_{r}}P_{k}*f\left(d_{jk}\right)}$$

(3) $$A_{i,2}=\sum_{r=1\sim h}\underset{j\in D_{r}}{\sum }\frac{S_{j}*K_{ij}*f\left(d_{ij}\right)}{\sum_{r=1\sim h}\sum_{k\in D_{r}}P_{k}*K_{jk}*f\left(d_{jk}\right)}$$

(4) $$A_{i,3}=\sum_{r=1\sim h}\underset{j\in D_{r}}{\sum }\frac{S_{j}*V_{ij}*f\left(d_{ij}\right)}{\sum_{r=1\sim h}\sum_{k\in D_{r}}P_{k}*V_{jk}*f\left(d_{jk}\right)}$$

(5) $$A_{i,4}=\sum_{r=1\sim h}\underset{j\in D_{r}}{\sum }\frac{S_{j}*K_{ij}^{V}*f\left(d_{ij}\right)}{\sum_{r=1\sim h}\sum_{k\in D_{r}}P_{k}*K_{jk}^{V}*f\left(d_{jk}\right)}$$

where *A_i,1_* is the simplest calculation of the geographical accessibility score of a location at demand *i* and implies the average amount of supply point resources enjoyed by people at demand in the location at demand *i*; *S_j_* represents the scale of supply at each service point (physiatrist) *j*; *P_k_* represents the size of the elderly population in the location at demand *k*; *d_ij_* is the route distance between the location at demand *i* and the service point *j*; *d_jk_* is the route distance between the service point *j* and the location at demand *k*. In the equations, *f(d_ij_)* is the distance-decay function, while the search radii of resources in this study are divided into three districts (*r* = 1~3) according to the respective distance. The first district (*d_ij_* ≤ 3 km) is the area that the elderly can reach on foot in about an hour [24]. The second district (3 km < *d_ij_* ≤ 15 km) is the area that the elderly can reach by driving for about half an hour. The third district (15 km < *d_ij_* ≤ 30 km) is the area that the elderly can reach by driving for about an hour. *f(d_ij_)* is shown in Equation (6):

(6) $$\left(d_{ij}\right)=\left\{\begin{array}{c} \begin{array}{c} 1,\;\;\;\;\;\;\;\;\;d_{ij}\leq 3\mathrm{km}\\ \frac{3}{d_{ij}},\;\;3\mathrm{km}<d_{ij}\leq 15\mathrm{km}\\ \frac{15}{{\left(d_{ij}\right)}^{2}},\;15\mathrm{km}<d_{ij}\leq 30\mathrm{km}\; \end{array}\\ 0,\;\;\;\;\;\;\;\;\;d_{ij}>30\mathrm{km} \end{array}\right\}$$

*A_i,2_* calculates the geographical accessibility score of a location at demand *i* when *K_ij_*, which is the different choice probabilities of the population at demand to approach various nearby service points, is taken into consideration. With considerations of the scale of supply at service points *S_j_* and distance decay *d_ij_*, *K_ij_* represents the choice probabilities of the location at demand *i* to service point *j* and is expressed in Equation (7):

(7) $$K_{ij}=\sum_{r=1\sim h}\frac{S_{j}*f\left(d_{ij}\right)}{\sum_{k\in D_{r}}S_{k}*f\left(d_{ik}\right)}$$

*A_i,3_* is a new method of calculation introduced in this study, which calculates the geographical accessibility score of a location at demand *i* when *V_j_*, the overall rating of the location point *j* summited to Google by ordinary users, is taken into consideration. It represents the crucial decision basis of people when they choose to visit a particular service point in reality. With considerations on rating *V_j_* and distance decay *d_ij_*, *V_ij_* represents the choice probabilities of the location at demand *i* to service point *j* and is expressed in Equation (8):

(8) $$V_{ij}=\sum_{r=1\sim h}\frac{V_{j}*f\left(d_{ij}\right)}{\sum_{k\in D_{r}}V_{k}*f\left(d_{ik}\right)}$$

*A_i,4_* is another new method of calculation introduced in this study. It calculates the geographical accessibility score of a location at demand *i* while integrating the factors of rating *V_j_*, the scale of supply at service points *S_j_*, distance decay *d_ij_*, and the different choice probabilities of the population at demand to approach various nearby service points $K_{ij}^{V}$. $K_{ij}^{V}$ is expressed in Equation (9):

(9) $$K_{ij}^{V}=\sum_{r=1\sim h}\frac{V_{j}*S_{j}*f\left(d_{ij}\right)}{\sum_{k\in D_{r}}V_{k}*S_{k}*f\left(d_{ik}\right)}$$

The flow of calculation follows Equations (1)–(8). First, we calculated the service load to be provided by each service point of physiatrists to the three districts divided by the distances and within a 30-km search radius of resources (the service load = the total population at demand in towns within a 30-km search radius/scale of service at the particular service point). Finally, we calculated the accumulated service load provided by the service points of physiatrists to each weighted center point of people at demand in towns, while the service points are within a 30-km search radius of the weighted center point. In this way, the accessible ratio of resources at the service points of physiatrists to the population at demand in towns was obtained, which is held to be the geographical accessibility score. Table 1 shows the calculation equations used in this study to evaluate the geographic accessibility scores of physical rehabilitation resources.

### 2.3. Google Rating

In the era of Web 2.0, consumers increasingly rely on the rating mechanism of online service platforms as crucial factors for decision-making. The online rating mechanism has become an important asset in the digital “reputation” economy [33]. For example, people who prepare to choose a hotel put a high value on the review scores left by tourists on travel information websites Agoda and Tripadvisor.

In 1995, Taiwan implemented the National Health Insurance policy, which provides convenient medical services to citizens. People are free to choose from various hospitals when they look for treatments. Faced with the competition in the free market, hospitals have adopted business models of marketing and branding to attract patients. Hospital rating mechanisms on online platforms, where people provide reviews voluntarily and freely, have emerged as crucial sources of references for patients’ healthcare-seeking decisions. Broadly speaking, rating mechanisms include blogging, Facebook, YouTube, and Google’s rating mechanism for businesses. Among them, Google Rating is the rating mechanism that performs best in structuring consumers’ feelings and is the most recognized by the public [33,34,35]. Google Rating scores are divided into 1~5 points, representing evaluations ranging from least satisfied to most satisfied.

Based on the open competition in Taiwan’s medical market, the high degree of freedom enjoyed by people in seeking treatment, and the multiple choice factors in healthcare-seeking decisions, this study innovates and introduces new methods of calculating the geographic accessibility scores of physical rehabilitation resources, in which Google ratings for businesses is included as a choice factor in the calculation equations. The methods are detailed in the descriptions of methods A3 and A4 or Equations (7) and (8).

### 2.4. Gini Coefficient

The Gini coefficient was defined by Italian statistician Corrado Gini based on the Lorenz curve as a measure of income distribution equality within a society [36]. The Gini coefficient can range from 1 to 0, wherein 1 represents complete inequality in people’s annual income distribution and 0 represents complete equality in income distribution. Generally speaking, a Gini coefficient below 0.2 indicates highly equitable income distribution, 0.2–0.3 represents equitable income distribution, 0.3–0.4 indicates bearable inequitable income distribution, 0.4–0.6 tends toward serious inequality in income distribution, and above 0.6 indicates high inequality in income distribution [37]. Therefore, when the Gini coefficient is above 0.6, the ruling authority would usually be advised to be on the alert for excessive income inequality within the society, as the situation may lead to social conflicts. Due to its nature, the Gini coefficient is also called the inequality coefficient. With reference to the above-mentioned scaling of the coefficient, this study explains the disparity in the accessible ratio of resources at service points of physiatrists to the population at demand in counties/cities.

The Gini coefficient was used in this study to evaluate the equality of the accessible ratio of service point resources to the population at demand. Therefore, a higher Gini coefficient in a county/city represents a more inequitable distribution of resources at service points to the population at demand. Based on the definition of *y*_1_ = *f*(*x*) of the Lorenz curve, the *y*-axis measures the accumulated percentage of the accessible ratio of service point resources in each town, while the *x*-axis measures the accumulated percentage of the population at demand in each town. The Gini coefficient is equal to the area between curve *y*_1_ and line *y*_2_, divided by the area below line *y*_2_. The Equation (10) is as follows [38]:

(10) $$G=\frac{\int_{0}^{1}\left(y_{2}-y_{1}\right)dx}{\int_{0}^{1}y_{2}dx}=\frac{\int_{0}^{1}\left(x-f\left(x\right)\right)dx}{\int_{0}^{1}xdx}=2\int_{0}^{1}\left(x-f\left(x\right)\right)dx$$

## 3. Results

### 3.1. Distribution of People at Demand in Towns and Physical Rehabilitation Resources

Table 2 summarizes the results of resource assessments using the regional average method (method A0). The National Health Insurance of Taiwan adopts an ideology of open and free competition concerning the setting up of hospitals. As a result, operating in metropolitan areas to attract clients is the first choice of most physical rehabilitation clinics and physiatrists. Furthermore, teaching hospitals focusing on physical rehabilitation training tend to cluster in metropolitan areas. Consequently, many physiatrists choose to register and practice in the same metropolitan area where they complete their specialist trainings. Therefore, in the six most urbanized municipalities (Taipei City, Kaohsiung City, New Taipei City, Taichung City, Tainan City, and Taoyuan City) where 68.12% of the elderly population live, the density of physiatrists per 10,000 elderly people ranged between 1.55% and 5.29%, while the average density of physiatrists on the main island of Taiwan was 1.80%.

### 3.2. Overview of the Google Ratings of Physical Rehabilitation Hospitals in Towns

Table 3 shows the Google ratings of physical rehabilitation hospitals in towns. Concerning mean values, eight counties/cities had a mean value lower than Taiwan’s average value. The eight counties/cities are Hsinchu County, Miaoli County, Yunlin County, Chiayi County, Pingtung County, Chiayi City, New Taipei City, and Taoyuan City. Among them, New Taipei City and Taoyuan City are densely populated and highly urbanized. The two municipalities also have a high number of hospitals and a physiatrist to elderly population ratio higher than Taiwan’s average value. However, the mean values of physical rehabilitation institutes’ Google ratings in the two municipalities are lower than Taiwan’s average value, while the standard deviations are higher than Taiwan’s average value. In the digital era, people rely heavily on the rating mechanism of online service platforms as crucial factors for decision-making, and the ratings can alter patients’ preference in seeking treatment. They may be more inclined to choose hospitals that have high ratings but greater travel distance comparatively. Therefore, as an innovation, this study introduced methods A3 and A4, which integrated Google ratings into the calculation of choice probabilities affecting geographic accessibility.

Some conventional inequality measures are the mean, median, Gini coefficient, maximum and minimum values [39]. The median is the middle number in a sorted list of numbers, with the same amount of numbers below and above. The median is sometimes used as opposed to the mean when there are outliers in a sequence that might skew the average of the values. The median of a sequence can be less affected by outliers than the mean. As physical rehabilitation resources are unequal in Taiwan, especially between urban and rural districts, this study applied these inequality measures to compare accessibility values between methods. Table 4 shows the comparison of all resulting scores of geographic accessibilities. All equations generated a minimum value (Min) of 0, which means that regardless of the calculation method chosen, there exist situations in which no physical rehabilitation resources are reachable within 30 km. The mean value of the results of the regional average method (method A0) is the lowest, but its value of standard deviation and maximum value are the highest among all methods. As the regional average method completely disregards the effectiveness of distance and sets limits on the cross-district usage of resources, the results of the regional average method create an illusion wherein the dispersion of geographic accessibility scores is the highest, with a median at 0 and the highest Gini coefficient.

In the results of the two-step floating catchment area method (method A1), the dispersion of geographic accessibility scores is the lowest, with the highest median and the lowest Gini coefficient. Method A2 represents the results of the traditional three-step floating catchment area method with consideration of choice probabilities related to distances and resource sizes; method A3 represents the results of the new three-step floating catchment area method with considerations of choice probabilities related to distances and Google ratings, and method A4 represents the results of the new three-step floating catchment area method with comprehensive considerations of distances, resource sizes, and Google ratings. The results in Table 4 show that, when comparing the results of methods A2–A4, which considered the choice probabilities of people’s healthcare-seeking behavior, and the results of method A1, which disregarded choice probabilities, the results of the former group show higher mean values, standard deviations, and maximum values and had lower median values. The results of methods A2–A4 also show higher dispersions of geographic accessibility scores and higher Gini coefficients.

When comparing the scores of geographic accessibilities in methods A2–A4, which considered the choice probabilities of people’s healthcare-seeking behavior, the values generated from method A4 show a tendency to land between the values of methods A3 and A2. With consideration of distance decays, sizes of hospitals, and Google ratings, the standard deviation and maximum value of the results of method A4 are lower than method A3, while the median value is higher than method A3. The dispersion of geographic accessibility scores and the Gini coefficient of method A4 are lower than method A3. It implies that concurrent consideration of non-spatial factors (online ratings and sizes of resource) are in better accordance with people’s decision-making behavior when they determine which resources of physical rehabilitation to use compared with the sole consideration of online rating factors.

### 3.3. Assessment of Distribution Inequality of People at Demand in Towns and Physical Rehabilitation Resources

Table 5 shows the mean values, standard deviations, and median values of geographic accessibility scores of counties/cities, which were evaluated by different calculation methods, while the differences in the resulting values are presented. Method 3 considers distances and Google ratings, whereas method A4 adds resource size factors to the basis of method 3. When the median values and mean values are compared, if the median value is lower than the mean value in a county/city, it implies that more than 50% of the resources have low accessibilities. In methods A3 and A4, only three counties/cities (Hsinchu City, New Taipei City, and Taoyuan City) have median values higher than mean values, which implies that the majority of the towns in the counties/cities enjoy plentiful resources. When we take the next step and compare the median values, 12 counties/cities have higher geographic accessibility scores in method A4 than in method A3, which are marked with “*” next to the median values of method 4. Among the counties/cities with more than 30 towns, Kaohsiung City and Pingtung County have higher accessibility scores in method A4, which means that in counties/cities with vast administrative regions, the resources of medical services are more inclined to concentrate in densely populated areas. Therefore, the people may have access to better medical services, as they would consider the credibility and service sizes of the hospitals and choose to visit hospitals that are farther but larger in size. The median values of method A4 rise due to the above reasons.

## 4. Discussion

Table 6 compares the regional average method (A0), the two-step floating catchment area method (A1), and the innovative three-step floating catchment area method A4 introduced in this study by the values of “median value minus mean value” and Gini coefficients. When the value of “median value minus mean value” of a county/city is negative, it implies that 50% of the medical resources in its towns have low accessibilities distribution. In addition, the mean values of the counties/cities that are lower than Taiwan’s average are marked with “*”. In the regional average method (A0), the counties/cities with low accessibilities in 50% of the medical resources in its towns are entirely different from those in methods A1 and A4. When we take a further step and compare the degree of inequality in resource accessibilities of counties/cities using the Gini coefficient, we can see that when we carry out an assessment with the regional average method (A0), as the analysis only included the amount of physical rehabilitation resources within the respective administrative regions, it led to calculation results in which 11 counties/cities fell into the category of resource distribution inequality. When the government allocates resources with reference to the regional average method (A0), it is easy to neglect the effects of distance and cross-district usage of services, and the phenomenon of resource distribution inequality worsens as a result. In method A4, Taitung County is the only place with a negative value of “median value minus mean value” and has a Gini coefficient that represents median inequality. The county belongs to Eastern Taiwan and comprises 14 towns. Despite the vast administrative region, there are only six hospitals and 14 board-certificated physiatrists operating in the county, which makes it the county with the highest inequality in resource accessibilities.

**Figure 1:**
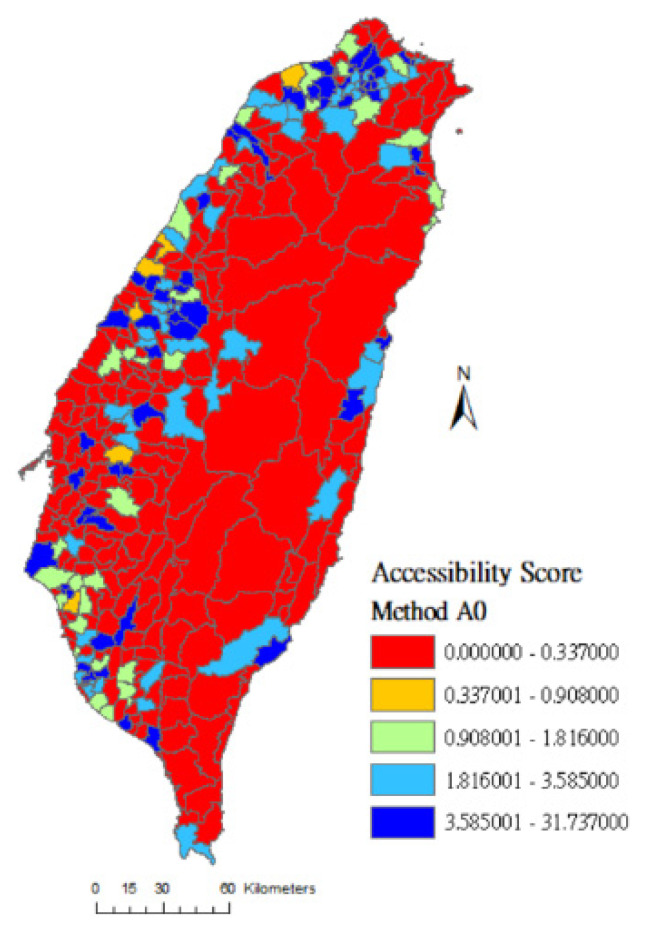
Accessibility score of rehabilitation physician service in Taiwan using method A0.

**Figure 2:**
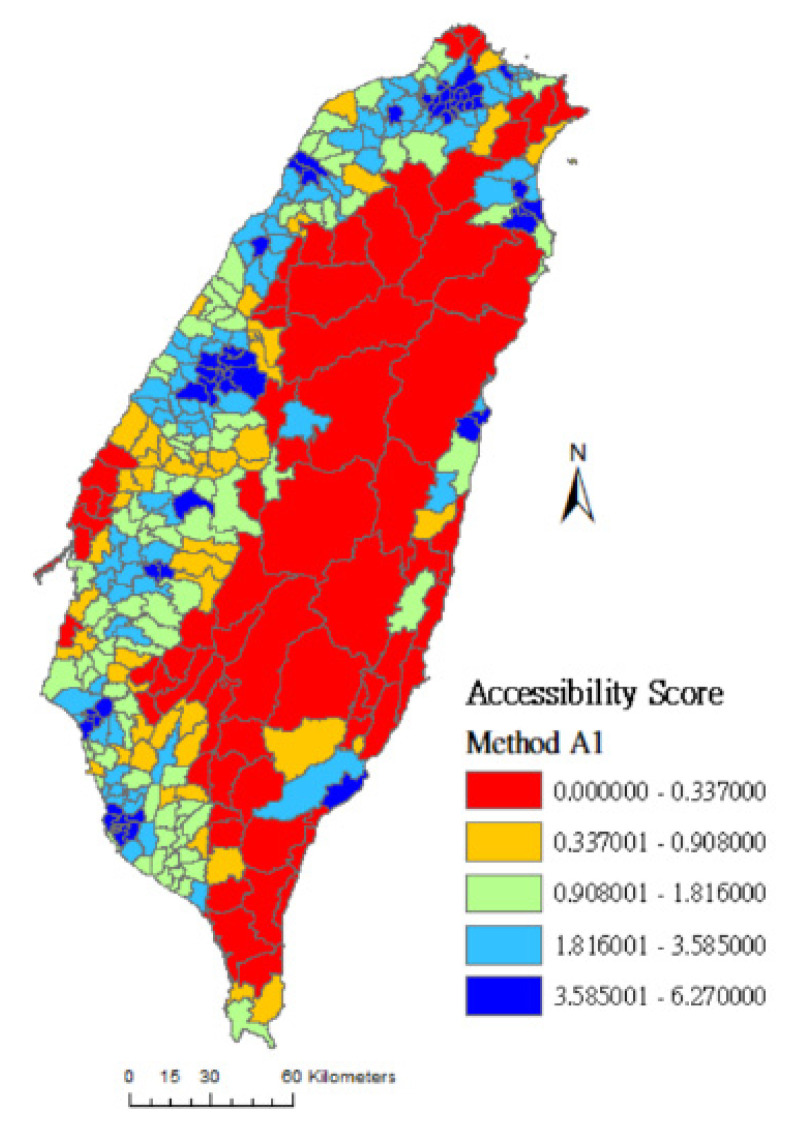
Accessibility score of rehabilitation physician service in Taiwan using method A1.

**Figure 3:**
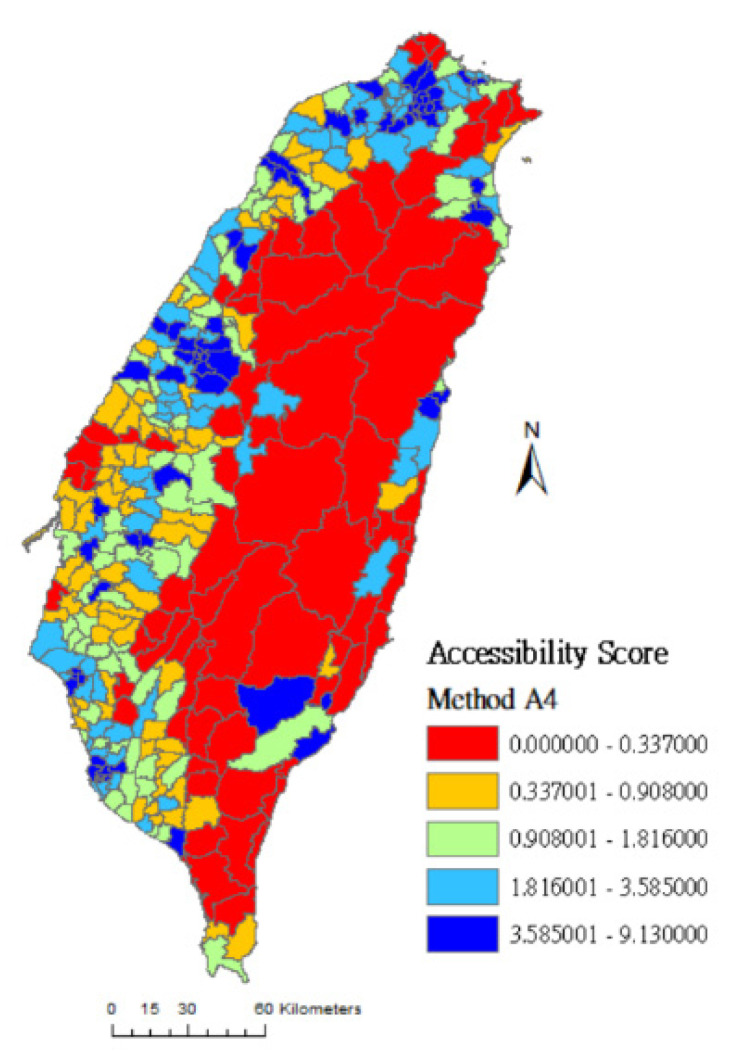
Accessibility score of rehabilitation physician service in Taiwan using method A4.

In this study, the geographical accessibility scores are grouped into quintiles and the spatial distributions of the accessibility scores of rehabilitation physicians are clearly presented on maps. The colors from lowest to highest accessibility score are red (0%~20%), orange (21%~40%), green (41%~60%), light blue (61%~80%), and dark blue (81%~100%). Figure 1 shows that red areas (low accessibility) measured by method A0 cover almost the entire island, meaning that many towns’ accessibility scores are evaluated as low because their medians are 0.0. Figure 2 and Figure 3 are drawn using method A1 and method A4, respectively. The difference between these two methods is that the latter considers the selection probability of each hospital. Figure 3 shows that the number of high-accessibility towns (light-blue and dark-blue area) is increased compared to Figure 2. This result shows that the distribution of medical service resources tends to concentrate in densely populated areas and downtowns. People may travel farther based on the reputation and service capacity of hospitals to get better medical services.

Based on the three-step floating catchment area method, Table 7 shows the results of assessments on inequality in resource accessibility with methods A2–A4. According to the calculation results of the three methods, 50% of the medical resources in towns had low accessibilities (negative value of “median value minus mean value”) in Taitung County and Hualien County. When we compared the inequality in resource accessibilities of counties/cities with the Gini coefficient, the value of Taitung County was close to the critical value of high inequality.

**Figure 4:**
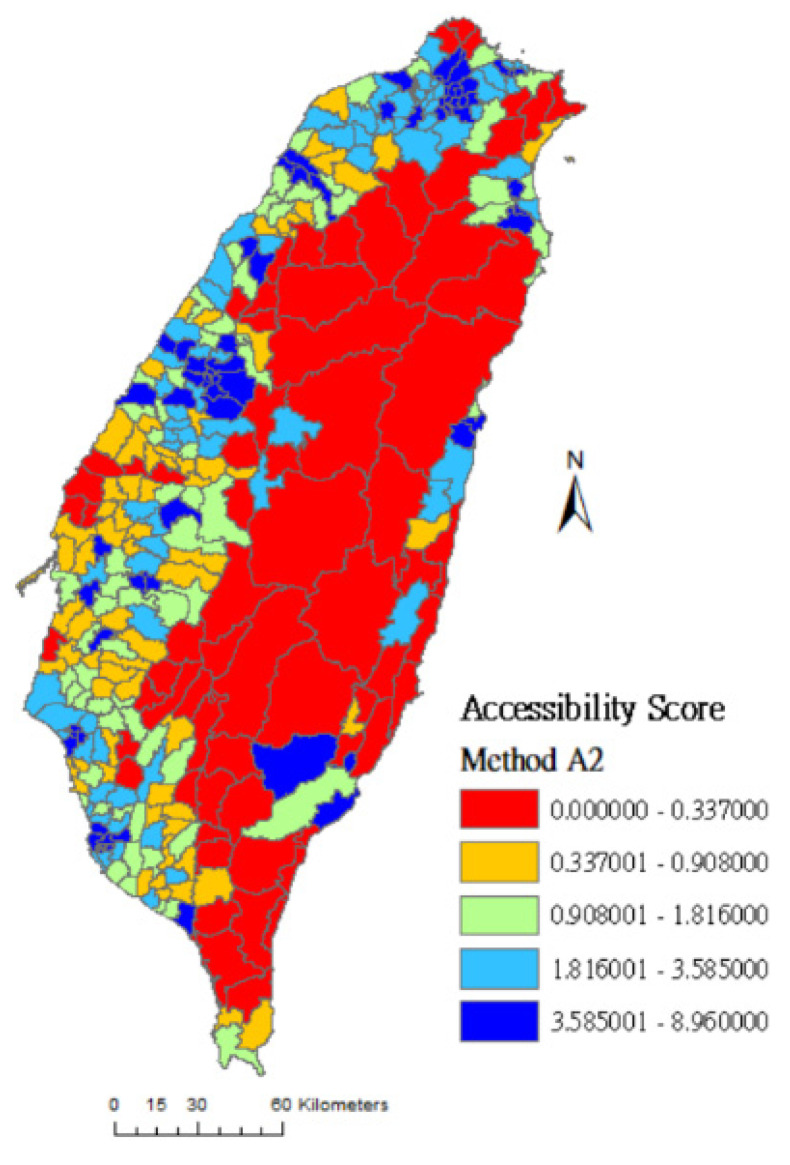
Accessibility score of rehabilitation physician service in Taiwan using method A2.

**Figure 5:**
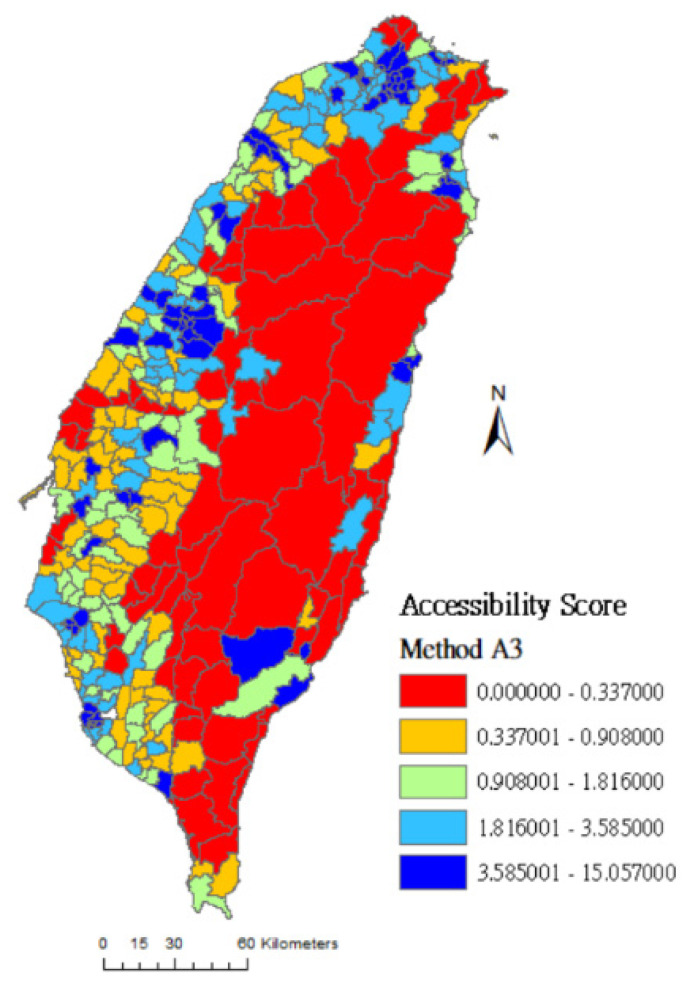
Accessibility score of rehabilitation physician service in Taiwan using method A3.

Comparing Figure 3, Figure 4 and Figure 5: Figure 3 was drawn by method A4 which considers both spatial factors (distance) and non-spatial factors (Google Rating score and resource capacity). In Figure 3, the number of dark-blue areas is increased in vast towns of the central and eastern administrative regions. Compared with method A2 (which only considers the selection probability of distance and resource capacity), and method A3 (which only considers the selection probability of distance and Google Rating score), the assessment result of method A4 may be more in line with people’s decision-making in choosing rehabilitation medical resources.

With method A4 proposed in this study, an assessment of physiatrist resource allocation policies on the main island of Taiwan was carried out. The results of our study have important implications for rehabilitation physician services and elderly care policy in Taiwan. In the first stage, the improvement of resources in Taitung County should be prioritized. The next in line should be the three counties/cities (Taitung County, Changhua County and Hualien County) where 50% of the medical resources in towns had low accessibilities and scored lower than Taiwan’s average. In the third stage, work should be carried out on the 12 counties/cities with low accessibilities in 50% of the medical resources in towns.

## 5. Conclusions

As the free market influences the medical environment, people have many choices of medical services and have access to duplicate medical treatments. Therefore, when we discuss the distributional fairness of physical rehabilitation resources, not only do we focus on the degree of coordination between the population at demand and service supply, but we also have to consider the distance factor when the patients travel to hospitals. In the information age, the rating mechanism of online service platforms, which allows ordinary people to review freely, has become a crucial source of references for people when they choose from numerous hospitals. In this study, various methods were utilized to assess the geographic accessibility of resources, while Google ratings were added as a choice factor for when people examine the credibility of hospitals. These features were integrated into method A4, which is an innovative research method to assess the appropriateness in the demand and supply of physical rehabilitation resources. Method A4 combines the spatial condition of travel distance, non-spatial conditions of hospital capacity, and Google Rating mechanism. This helps examine whether the allocation of physical rehabilitation resources shows inequality due to regional differences.

With restrictions on the access of data and lack of details, the limitations encountered in this study include the following: (1) The people at demand were positioned at the weighted center points of population in geometry. This only provides reference locations of the people at demand and cannot reflect the exact locations of each elderly person at demand in reality. The author suggests employing finer space scales such as the scale of basic statistical areas (BSAs) for better research in the future. (2) Activity areas regarding geographical accessibility were merely represented by route distances and the estimation of the range of activities of the elderly may not be precise. In the future, transportation time or different vehicles can be integrated into calculations and evaluations. (3) This study only examines geographical accessibility. Relevant social and economic conditions can be weighted and added to the calculation processes in the future to facilitate analysis combined with geographical accessibility. (4) The open data of the government do not disclose the number of users, statistics of service items, and details of duplicate medical treatments in hospitals. Therefore, concerning the differences in people at demand and the actual number of users, a cross-validation cannot be carried out in this study. At the same time, assessments and comparisons between the service loads and the actual service effectiveness of hospitals cannot be carried out.

## Figures and Tables

**Table 1:** Definition of elderly physical rehabilitation resources geographic accessibility scores.

Method	Description	Equation	Distance-Decay Function
A0	Regional average method	$A_{i,0}=\frac{\sum_{j\in D_{i}}S_{j}}{P_{i}\;}$	1
A1	Two-step floating catchment area method without choice probability	$A_{i,1}=\sum_{r=1\sim h}\underset{j\in D_{r}}{\sum }\frac{S_{j}*f\left(d_{ij}\right)}{\sum_{r=1\sim h}\sum_{k\in D_{r}}P_{k}*f\left(d_{jk}\right)}$	$f\left(d_{ij}\right)=\left\{\begin{array}{c} \begin{array}{c} 1,\;\;\;\;\;\;\;\;\;d_{ij}\leq 3\mathrm{km}\\ \frac{3}{d_{ij}},\;\;\;3\mathrm{km}<d_{ij}\leq 15\mathrm{km}\\ \frac{15}{{\left(d_{ij}\right)}^{2}},\;15\mathrm{km}<d_{ij}\leq 30\mathrm{km}\; \end{array}\\ 0,\;\;\;\;\;\;\;\;\;d_{ij}>30\mathrm{km} \end{array}\right\}$
A2	Three-step floating catchment area method with considerations of choice probability *K_ij_*	$A_{i,2}=\sum_{r=1\sim h}\underset{j\in D_{r}}{\sum }\frac{S_{j}*K_{ij}*f\left(d_{ij}\right)}{\sum_{r=1\sim h}\sum_{k\in D_{r}}P_{k}*K_{jk}*f\left(d_{jk}\right)}$	
A3	Three-step floating catchment area method with considerations of choice probability *V_ij_*	$A_{i,3}=\sum_{r=1\sim h}\underset{j\in D_{r}}{\sum }\frac{S_{j}*V_{ij}*f\left(d_{ij}\right)}{\sum_{r=1\sim h}\sum_{k\in D_{r}}P_{k}*V_{jk}*f\left(d_{jk}\right)}$	
A4	Three-step floating catchment area method with considerations of choice probability $K_{ij}^{V}$	$A_{i,4}=\sum_{r=1\sim h}\underset{j\in D_{r}}{\sum }\frac{S_{j}*K_{ij}^{V}*f\left(d_{ij}\right)}{\sum_{r=1\sim h}\sum_{k\in D_{r}}P_{k}*K_{jk}^{V}*f\left(d_{jk}\right)}$	

**Table 2:** Summary statistics of 65+ population and physical rehabilitation physicians’ scores by administrative districts (method A0).

Administrative District	65^+^ Population	65^+^ Population %	Number of Towns	Number of Physicians	Physicians-to 10,000 Population %
Yilan County	76,134	2.10%	12	25	1.82
Hsinchu County	71,911	1.99%	13	16	0.91
Miaoli County	91,283	2.52%	18	21	1.13
Changhua County	205,532	5.68%	26	42	0.99
Nantou County	89,157	2.46%	13	12	0.79
Yunlin County	127,220	3.52%	20	19	0.81
Chiayi County	99,858	2.76%	18	13	0.92
Pingtung County	140,607	3.89%	32	18	0.65
Taitung County	35,707	0.99%	14	8	0.54
Hualien County	55,009	1.52%	13	20	1.49
Keelung City	62,020	1.71%	7	23	3.35
Hsinchu City	57,138	1.58%	3	22	3.29
Chiayi City	42,062	1.16%	2	24	5.72
Taipei City	483,523	13.36%	12	255	5.29
Kaohsiung City	444,875	12.29%	38	143	2.52
New Taipei City	590,644	16.32%	29	172	2.09
Taichung City	368,586	10.19%	29	141	3.66
Tainan City	299,640	8.28%	37	83	1.55
Taoyuan City	277,972	7.68%	13	83	2.19
Total	3,618,878	100%	349	1140	1.80

**Table 3:** Summary statistics of physical rehabilitation hospitals’ Google rating.

Administrative District	Number of Hospitals	Mean		SD		Min		Max	
Yilan County	15	3.71		0.74	◎	2.40	*	5.00	
Hsinchu County	13	3.65	*	0.47		2.60		4.60	*
Miaoli County	12	3.49	*	0.63		2.50	*	4.80	*
Changhua County	23	3.75		0.70	◎	2.50	*	5.00	
Nantou County	11	3.87		0.86	◎	2.70		5.00	
Yunlin County	11	3.69	*	0.57		3.00		4.90	
Chiayi County	5	3.64	*	0.21		3.40		3.90	*
Pingtung County	15	3.41	*	0.58		2.50	*	4.30	*
Taitung County	6	3.78		0.77	◎	2.50	*	4.90	
Hualien County	11	3.76		0.80	◎	2.10	*	5.00	
Keelung City	11	3.85		0.55		2.80		4.50	*
Hsinchu City	12	3.79		0.67	◎	3.10		4.90	
Chiayi City	11	3.66	*	0.49		3.20		4.50	*
Taipei City	114	3.79		0.56		2.60		5.00	
Kaohsiung City	96	3.78		0.68	◎	2.20	*	5.00	
New Taipei City	102	3.62	*	0.72	◎	1.90	*	5.00	
Taichung City	83	3.75		0.68	◎	2.50	*	5.00	
Tainan City	57	3.74		0.69	◎	2.20	*	5.00	
Taoyuan City	50	3.63	*	0.70	◎	2.00	*	4.90	
Total	658								
Average		3.72		0.66		2.56		4.80	

Note: 1. *: lower than average. 2. ◎: higher than average.

**Table 4:** Summary statistics of physical rehabilitation resources accessibility scores by methods A0–A4.

Method	Mean	Median	SD	Min	Max	Median-Mean	Gini Coefficient
A0	1.80	0.00	3.32	0.00	31.74	−1.80	0.53
A1	1.87	1.56	1.58	0.00	6.27	−0.31	0.05
A2	1.89	1.20	1.72	0.00	8.96	−0.69	0.10
A3	1.91	1.16	1.96	0.00	15.06	−0.75	0.15
A4	1.90	1.21	1.74	0.00	9.13	−0.69	0.11

**Table 5:** Summary statistics of physical rehabilitation resources accessibility scores by methods A0–A4.

Administrative District	Number of Towns				Estimated by 10,000 * Capacity/People												
		Method A0			Method A1			Method A2			Method A3			Method A4			
		Mean	Median	SD	Mean	Median	SD	Mean	Median	SD	Mean	Median	SD	Mean	Median		SD
Yilan County	12	1.82	0.00	3.33	2.43	2.52	1.73	2.27	1.72	1.90	2.27	1.72	2.00	2.28	1.75	*	1.90
Hsinchu County	13	0.91	0.00	1.96	1.67	1.64	1.13	1.45	0.91	1.42	1.41	0.89	1.39	1.44	0.91	*	1.42
Miaoli County	18	1.13	0.00	1.79	1.57	1.66	1.34	1.45	1.10	1.51	1.44	1.08	1.51	1.44	1.08		1.50
Changhua County	26	0.99	0.00	1.78	1.61	1.06	0.96	1.49	0.75	1.25	1.46	0.72	1.27	1.49	0.75	*	1.25
Nantou County	13	0.79	0.00	1.06	0.83	0.81	0.74	0.92	0.50	0.94	0.89	0.51	0.90	0.92	0.52		0.93
Yunlin County	20	0.81	0.00	1.78	1.17	1.02	0.97	1.16	0.71	1.2	1.18	0.64	1.31	1.16	0.70	*	1.21
Chiayi County	18	0.92	0.00	3.02	1.59	1.64	1.05	1.33	0.95	1.11	1.30	0.85	1.48	1.33	0.95	*	1.11
Pingtung County	32	0.65	0.00	1.47	0.88	0.97	0.70	0.89	0.74	0.94	0.88	0.70	0.98	0.89	0.74	*	0.93
Taitung County	14	0.54	0.00	1.38	0.55	0.02	1.25	0.87	0.03	1.76	0.88	0.03	1.81	1.00	0.03		2.14
Hualien County	13	1.49	0.00	2.50	1.63	0.78	2.03	1.66	0.82	2.04	1.65	0.82	2.06	1.66	0.82		2.04
Keelung City	7	3.35	1.83	4.07	2.67	2.52	0.58	3.45	3.45	1.32	3.38	3.23	1.72	3.45	3.40	*	1.31
Hsinchu City	3	3.29	1.92	4.15	3.41	3.88	1.16	3.26	4.03	1.80	3.29	3.91	1.9	3.26	4.03	*	1.80
Chiayi City	2	5.72	5.72	0.34	4.21	4.21	0.12	4.64	4.64	0.07	4.59	4.59	0.22	4.64	4.64	*	0.06
Taipei City	12	5.29	5.43	2.45	4.74	5.00	0.86	4.73	4.66	0.44	4.84	4.67	0.63	4.70	4.64		0.43
Kaohsiung City	38	2.52	0.47	4.73	2.06	1.78	1.55	2.11	1.52	1.72	2.29	1.45	2.63	2.12	1.54	*	1.73
New Taipei City	29	2.09	1.43	3.09	2.08	2.08	1.70	2.30	2.48	2.00	2.37	2.48	2.69	2.31	2.44		2.03
Taichung City	29	3.66	2.52	6.09	3.13	2.83	1.99	3.10	3.03	1.87	3.15	2.88	1.96	3.10	3.03	*	1.89
Tainan City	37	1.55	0.00	2.93	1.68	1.28	1.41	1.67	1.00	1.51	1.65	1.01	1.54	1.67	0.99		1.51
Taoyuan City	13	2.19	2.34	1.72	2.13	2.00	1.18	2.30	2.58	1.24	2.24	2.38	1.22	2.29	2.55	*	1.24
Total	349																
Average		1.80	0.00	3.32	1.87	1.56	1.58	1.89	1.20	1.72	1.91	1.16	1.96	1.90	1.21	*	1.74

Note: *: (median by A3) − (median by A4) < 0.

**Table 6:** Measures of geographic inequality of physical rehabilitation resources accessibility scores by methods A0, A1, A4.

									Estimated by 10,000 * Capacity/People			
Administrative District	Method A0				Method A1				Method A4			
	Median-Mean		Gini Coefficient		Median-Mean		Gini Coefficient		Median-Mean		Gini Coefficient	
Yilan County	−1.82	*	0.49	◎	0.09		0.08		−0.53		0.11	
Hsinchu County	−0.91		0.45	◎	−0.03		0.10		−0.54		0.16	
Miaoli County	−1.13		0.39		0.09		0.23		−0.36		0.24	
Changhua County	−0.99		0.27		−0.55	*	0.38		−0.74	*	0.28	
Nantou County	−0.79		0.43	◎	−0.02		0.16		−0.40		0.25	
Yunlin County	−0.81		0.61	◎◎	−0.16		0.24		−0.46		0.29	
Chiayi County	−0.92		0.81	◎◎	0.06		0.18		−0.38		0.26	
Pingtung County	−0.65		0.70	◎◎	0.09		0.13		−0.16		0.15	
Taitung County	−0.54		0.20		−0.53	*	0.19		−0.97	*	0.59	◎
Hualien County	−1.49		0.30		−0.85	*	0.08		−0.84	*	0.09	
Keelung City	−1.51		0.47	◎	−0.14		0.02		−0.05		0.11	
Hsinchu City	−1.37		0.33		0.47		0.03		0.77		0.02	
Chiayi City	0.00		0.03		0.00		0.01		0.00		0.01	
Taipei City	0.14		0.22		0.27		0.09		−0.07		0.03	
Kaohsiung City	−2.05	*	0.63	◎◎	−0.28		0.03		−0.58		0.09	
New Taipei City	−0.66		0.48	◎	0.00		0.08		0.13		0.11	
Taichung City	−1.13		0.55	◎	−0.31		0.18		−0.08		0.20	
Tainan City	−1.55		0.59	◎	−0.40	*	0.05		−0.68		0.09	
Taoyuan City	0.15		0.14		−0.13		0.06		0.26		0.06	
Average	−1.80		0.53	◎	−0.31		0.05		−0.69		0.11	

Notes: 1. Level of distribution inequality estimated by score of “Median-Mean”. *: smaller than average. 2. Level of distribution inequality estimated by Gini coefficient. ◎: 0.4~0.6, median inequality, ◎◎: > 0.6, high inequality.

**Table 7:** Measures of geographic inequality of physical rehabilitation resources accessibility scores by methods A2–A4.

									Estimated by 10,000 * Capacity/People			
Administrative District	Method A2				Method A3				Method A4			
	Median-Mean		Gini Coefficient		Median-Mean		Gini Coefficient		Median-Mean		Gini Coefficient	
Yilan County	−0.55		0.12		−0.55		0.18		−0.53		0.11	
Hsinchu County	−0.53		0.16		−0.52		0.16		−0.54		0.16	
Miaoli County	−0.35		0.24		−0.35		0.24		−0.36		0.24	
Changhua County	−0.73	*	0.28		−0.74		0.26		−0.74	*	0.28	
Nantou County	−0.42		0.20		−0.38		0.25		−0.40		0.25	
Yunlin County	−0.45		0.29		−0.54		0.31		−0.46		0.29	
Chiayi County	−0.39		0.26		−0.44		0.31		−0.38		0.26	
Pingtung County	−0.15		0.15		−0.18		0.17		−0.16		0.15	
Taitung County	−0.84	*	0.54	◎	−0.85	*	0.55	◎	−0.97	*	0.59	◎
Hualien County	−0.84	*	0.09		−0.84	*	0.10		−0.84	*	0.09	
Keelung City	0.00		0.11		−0.14		0.14		−0.05		0.11	
Hsinchu City	0.77		0.02		0.62		0.03		0.77		0.02	
Chiayi City	0.00		0.01		0.00		0.00		0.00		0.01	
Taipei City	−0.08		0.03		−0.17		0.05		−0.07		0.03	
Kaohsiung City	−0.60		0.09		−0.83	*	0.21		−0.58		0.09	
New Taipei City	0.17		0.12		0.11		0.22		0.13		0.11	
Taichung City	−0.07		0.19		−0.27		0.23		−0.08		0.20	
Tainan City	−0.67		0.09		−0.63		0.10		−0.68		0.09	
Taoyuan City	0.28		0.07		0.14		0.07		0.26		0.06	
**Average**	**−0.69**		**0.10**		**−0.75**		**0.15**		**−0.69**		**0.11**	

Notes: 1. Level of distribution inequality estimated by score of “Median-Mean”. *: smaller than average. 2. Level of distribution inequality estimated by Gini coefficient. ◎: 0.4~0.6, median inequality.
